# Evolution of electrocardiographic manifestations mimicking acute coronary syndrome secondary to hypoparathyroidism: a case report

**DOI:** 10.3389/fcvm.2026.1932593

**Published:** 2026-09-03

**Authors:** Gerile Zhang, Riletu Ge, Jing Gao, Yuzhen Zhou

**Affiliations:** Department of Cardiology(2), Xing'an League People's Hospital, Ulanhot, Inner Mongolia, China

**Keywords:** a case report, acute coronary syndrome, chest pain, electrocardiographic evolution, hypoparathyroidism

## Abstract

**Objective:**

To promptly identify electrocardiographic (ECG) manifestations, including deep T-wave inversions and QT-interval prolongation, secondary to hypoparathyroidism and to improve its diagnostic accuracy and therapeutic management.

**Methods:**

We conducted a comprehensive analysis of the integrated treatment for a patient presenting with chest pain and evolving ECG changes secondary to hypoparathyroidism, supplemented by a review of the relevant literature.

**Results:**

In this case, a patient diagnosed with hypoparathyroidism presented with chest pain and dynamic ECG alterations. Following a confirmed diagnosis, treatment with calcium supplementation successfully alleviated the patient's chest pain and resulted in the complete normalization of the previously abnormal ECG findings.

**Conclusion:**

Clinical presentations mimicking angina pectoris, accompanied by secondary ECG changes such as deep T-wave inversions and QT-interval prolongation, are relatively uncommon in patients with hypoparathyroidism. A thorough evaluation combining clinical presentation, characteristic signs, and laboratory investigations can significantly reduce the rate of misdiagnosis.

## Introduction

Hypoparathyroidism is an endocrine disorder characterized by insufficient secretion of parathyroid hormone (PTH). This insufficiency, resulting from developmental failure or destruction of the parathyroid glands, leads to hypocalcemia and hyperphosphatemia, which in turn cause a series of clinical manifestations. The associated low calcium levels increase neuromuscular excitability, potentially inducing laryngospasm or respiratory muscle spasm. Patients may present with symptoms such as chest pain, chest tightness, shortness of breath, and a choking sensation in the throat. Concurrently, dynamic electrocardiographic (ECG) changes, including deep T-wave inversions and prolongation of the QT interval, are often observed. These clinical and electrocardiographic features can closely mimic those of acute coronary syndrome, leading to frequent misdiagnosis in clinical practice. This report details a case of a patient admitted to our cardiology department with hypoparathyroidism, whose presentation of chest pain and dynamic ECG evolution simulated acute coronary syndrome.

Informed consent was obtained from the patient for the use of all clinical information and data presented in this case report. The study protocol was reviewed and approved by the Ethics Committee of Xing'anmeng People's Hospital.

### Clinical data

**Figure 1:**
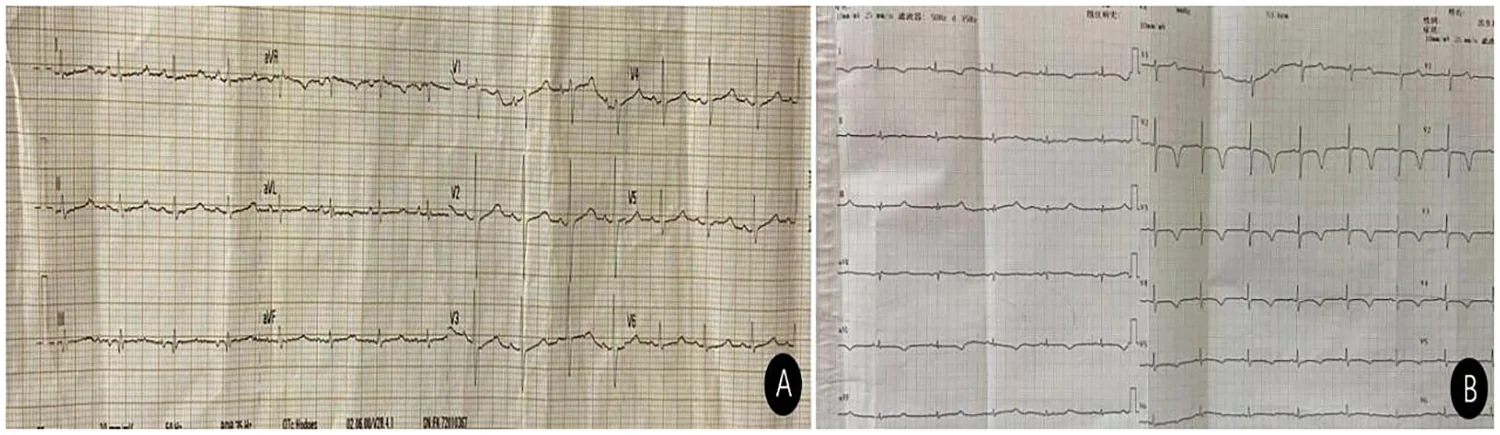
Emergency electrocardiograms. **(A)** Sinus rhythm, essentially normal tracing. **(B)** Sinus bradycardia with T-wave inversions (0.2–0.9 mV) in leads I, aVL, and V1–V5, and a QTc interval of 600 ms.

A 53-year-old male was admitted to the hospital with a chief complaint of “intermittent chest pain, chest tightness, and shortness of breath for 7 days, aggravated for 2 h.” Seven days prior to admission, the patient began experiencing retrosternal chest pain without an obvious precipitating factor. The pain was described as a dull, oppressive sensation accompanied by chest tightness and shortness of breath. There was no associated fever, chills, cough, or sputum production. Each episode lasted approximately 3–5 min and resolved spontaneously. The patient sought evaluation in the emergency department at that time. Initial laboratory workup showed normal results for cardiac enzymes, troponin, and complete blood count. However, the serum potassium level was low at 3.2 mmol/L. An electrocardiogram (ECG) revealed normal sinus rhythm and was interpreted as essentially normal (Figure 1A). A chest CT scan showed multiple linear opacities in both lungs and several micronodules in the left lung. The patient was prescribed oral potassium citrate granules for potassium repletion but did not undergo further systematic evaluation. His symptoms continued to occur intermittently but remained tolerable.

Two hours before admission, the patient experienced a significant exacerbation of his retrosternal pain, now accompanied by pronounced chest tightness, shortness of breath, and a choking sensation in the throat. He returned to the emergency department. A repeat ECG demonstrated sinus bradycardia (ventricular rate: 53 bpm) with T-wave inversions (0.2–0.9 mV) in leads I, aVL, and V1–V5 and a markedly prolonged corrected QT interval (QTc) of 600 ms (Figure 1B). Emergency treatment included intravenous nitroglycerin for coronary vasodilation and tramadol for analgesia. Following this, he was admitted to the cardiology department with a provisional diagnosis of Acute Coronary Syndrome (ACS).

### Past medical history

The patient denied any significant family or personal history of genetic diseases or hypocalcemia. His medical history included a 23-year history of liver cirrhosis, managed intermittently with “Liver Protectant Tablets” without systematic follow-up. Thyroid nodules had been identified two years prior, with normal thyroid function and no specific intervention. He had undergone cholecystectomy ten years earlier for gallstone pancreatitis.

### Physical examination on admission

Vital signs were as follows: temperature 36.2°C, heart rate 75 bpm, respiratory rate 19 breaths per minute, and blood pressure 147/110 mmHg. The patient presented with an acute, distressed facial expression but was alert and responsive. Breath sounds were clear bilaterally with no audible rales or rhonchi. The cardiac examination revealed a normal heart border, a regular rhythm at 75 bpm, and the absence of murmurs. There was no edema in the lower extremities. All physiological reflexes were present, and pathological reflexes were not elicited.

#### Laboratory findings

Upon admission, complete blood count, myoglobin, troponin, coagulation profile, random blood glucose, serum electrolytes (sodium, chloride, potassium), and liver/kidney function tests were largely unremarkable, with the following exceptions: creatine kinase (CK) 875.5 U/L (reference 50–310), CK-MB 15.1 U/L (0–25), *α*-hydroxybutyrate dehydrogenase 269.6 U/L, lactate dehydrogenase 530.7 U/L, NT-pro-BNP 213.1 pg/mL (0–125), serum calcium 1.44 mmol/L (2.11–2.52), D-dimer 1.96 mg/L (0–0.55), and C-reactive protein 16.02 mg/L (0–10). Arterial blood gas analysis showed: pH 7.5, PCO_2_ 31 mmHg, PO_2_ 138 mmHg, and O_2_ saturation 99%.

#### Ancillary investigations

A follow-up ECG on the cardiology ward revealed sinus rhythm, T-wave inversions (0.2–0.7 mV) in leads I, aVL, and V1–V6, QTc 518 ms, at a rate of 75 bpm. Echocardiography showed no structural abnormalities, with mildly reduced left ventricular diastolic function and LVEF of 54%. Chest CT demonstrated minor consolidation in the lower lobes of both lungs and a small left pleural effusion.

### Admission diagnoses

1. Acute coronary syndrome
2. Thyroid nodule
3. Liver cirrhosis
4. Pneumonia
5. Small left pleural effusion

### Diagnostic and therapeutic course

Following admission to the cardiology department, the patient's presentation—encompassing chest pain, tightness, shortness of breath, a choking sensation, and diaphoresis—combined with dynamic ECG changes and abnormal cardiac enzyme levels led to an initial diagnosis of Acute Coronary Syndrome (ACS), with suspicion of significant coronary artery disease. After detailed discussion with the patient and his family, an emergency coronary angiography was performed.

**Figure 2:**
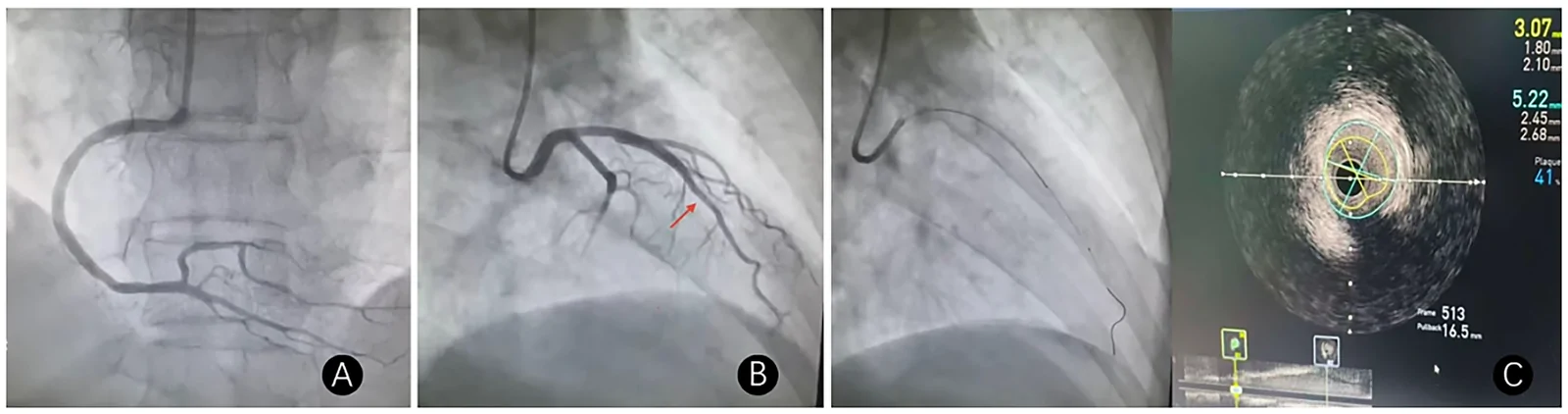
Coronary angiography and intravascular ultrasound (IVUS) findings. **(A)** No significant stenosis observed in the right coronary artery. **(B)** Coronary angiography showing 50% stenosis in the mid-segment of the left anterior descending (LAD) artery, with a suspected thrombus shadow. **(C)** IVUS examination revealing a minimum lumen area of 3.07 mm2 and a plaque burden of 41% in the mid-LAD segment.

The angiographic findings revealed a 50% stenosis in the mid-segment of the left anterior descending artery (LAD) with a suspected thrombus shadow (Figure 2A). The right coronary artery showed no significant stenosis (Figure 2B). Subsequent intravascular ultrasound (IVUS) evaluation of the LAD indicated a minimum lumen area of 3.07 mm^2^ with a plaque burden of 41% (Figure 2C). As the IVUS examination ruled out acute thrombus or a critically severe stenotic lesion, the procedure was concluded, and the patient was transferred back to the ward in stable condition.

However, the patient continued to experience recurrent episodes of chest pain, tightness, shortness of breath, throat constriction, and now also reported new symptoms of facial and limb paresthesia (numbness and tingling). An urgent serum calcium test returned a critically low result of 1.44 mmol/L. Immediate intravenous infusion of 2 g calcium gluconate provided marked relief of all the aforementioned symptoms.

Given the profound hypocalcemia,further laboratory investigations were pursued. Results showed normal levels of thyroid-stimulating hormone, vitamin D, and urinary calcium. However, serum inorganic phosphate was elevated at 1.65 mmol/L (reference: 0.85–1.51 mmol/L), and parathyroid hormone (PTH) was low at 7.4 pg/mL (reference: 15–68.3 pg/mL). Serum magnesium was within normal range at 0.86 mmol/L.Based on this biochemical profile(hypocalcemia,hyperphosphatemia, and low PTH), a supplemental diagnosis of Hypoparathyroidism was established.

The calcium replacement regimen was adjusted accordingly: intravenous calcium gluconate (2 g, twice daily) and oral vitamin D₂ soft capsules (0.25 mg, once daily). Following an endocrinology consultation, the oral therapy was further optimized to Calcium Carbonate and Vitamin D₃ Tablets (600 mg, twice daily after meals) and Calcitriol (1,25-dihydroxyvitamin D₃, 0.25 µg once daily).

**Figure 3:**
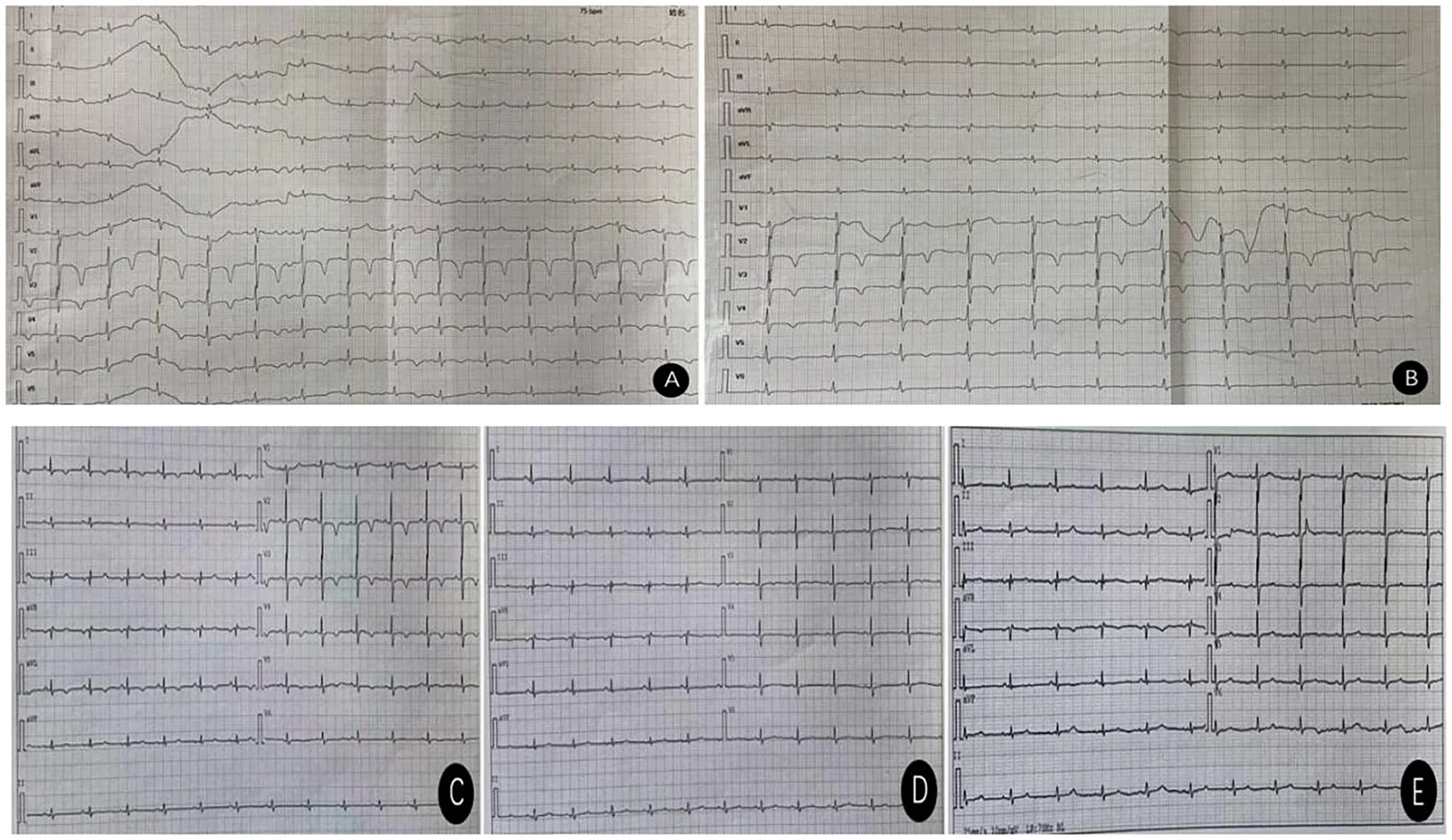
Serial electrocardiograms during hospitalization. **(A)** Day 1: sinus rhythm, with T-wave inversions (0.2–0.7 mV) in leads I, aVL, and V1–V6, and a QTc interval of 518 ms. **(B)** Day 2: sinus rhythm, with T-wave inversions (0.15–0.5 mV) in the same leads, and a QTc interval of 492 ms. **(C)** Day 3: sinus rhythm, with T-wave inversions (0.1–0.4 mV) in the same leads, and a QTc interval of 478 ms. **(D)** Day 5: sinus rhythm, with flattened T waves in the same leads, and a QTc interval of 457 ms. **(E)** Day 7: sinus rhythm, essentially normal electrocardiogram(QTc440 ms).

With the above treatment, the patient's symptoms—including chest pain, chest tightness, shortness of breath, throat constriction, and facial/limb paresthesia—completely resolved. Serial ECGs demonstrated gradual normalization of the previously observed deep T-wave inversions and QT-interval prolongation over the subsequent days (ECGs from hospital days 1, 2, 3, 5, and 7 are shown in Figure 3). Crucially, comprehensive cardiac workup—including serial cardiac enzymes/troponin (Table 1), echocardiography, and coronary angiography—did not support a diagnosis of acute myocardial infarction.

**Table 1:** Serial laboratory parameters during hospitalization.

Parameter	Day1(Admission Day)	Day 2	Day 3	Day 5	Day 7	Reference range
QTc interval (ms)	518	492	478	457	440	<450
LDH (U/L)	530.7	420.3	338.9	295.2	240.2	120–250
HBDH (U/L)	269.6	214.5	189.6	169.1	147.3	71–182
CK (U/L)	875.5	683.6	421.7	122.2	107.5	50–310
CK-MB (U/L)	15.1	13.2	12.4	11.4	8.2	0–25
Troponin (ng/ml)	0.005	–	0.005	–	–	0–0.014
Serum Calcium (mmol/L)	1.44	1.44	1.92	1.82	2.02	2.11–2.51
Serum inorganic phosphate (mmol/L)	–	1.65	–	–	–	0.85–1.51
Serum Potassium (mmol/L)	4.00	5.15	3.89	3.87	3.80	3.5–5.3

The patient's angina-like symptoms and dynamic ECG changes were attributed to hypocalcemia secondary to hypoparathyroidism. The mechanism involved hypocalcemia-induced increased neuromuscular excitability, potentially causing laryngospasm or respiratory muscle spasm (manifesting as chest pain, tightness, shortness of breath, and throat constriction) and affecting cardiac repolarization (manifesting as deep T-wave inversions and QT prolongation). Therefore, the initial diagnosis of ACS was excluded and revised to Hypoparathyroidism. The patient had a prior history of thyroid nodules. Further thyroid ultrasound revealed no significant abnormalities in the parathyroid region, while a relatively large nodule (approximately 0.85 ×  0.77 cm) was identified in the left lobe of the thyroid, classified as C-TIRADS-4A.(C-TIRADS denotes China-TIRADS, the Chinese Thyroid Imaging Reporting and Data System recommended in the 2020 Chinese Medical Association Ultrasound Guidelines; it serves as a risk-stratification system for thyroid nodules in China). A fine-needle aspiration biopsy of the thyroid nodule performed at an external hospital indicated a follicular neoplasm (Bethesda category IV).

## Discussion

Hypoparathyroidism (HP) is an endocrine disorder resulting from insufficient secretion of parathyroid hormone (PTH), leading to hypocalcemia and hyperphosphatemia, which can affect multiple organ systems including the cardiovascular, central nervous, respiratory, and musculoskeletal systems. Internationally, HP is classified as a rare disease. Epidemiological data are limited, but chronic HP prevalence is estimated between 6.4 and 37 per 100,000, with an incidence of 0.8 to 2.3 per 100,000 person-years. Non-surgical HP is even rarer, with a global prevalence estimated between 0.7 and 8 per 100,000. The primary cause of HP is iatrogenic damage to the parathyroid glands during neck surgery (>75% of cases). Other etiologies include autoimmune destruction, genetic developmental anomalies, infiltrative diseases (e.g., Wilson's disease, hemochromatosis), and compression or invasion by neck masses. Notably, decompensated liver cirrhosis and severe hypomagnesemia can suppress PTH secretion and induce peripheral PTH resistance, resulting in functional PTH insufficiency; these are reversible upon correcting the underlying condition and do not constitute structural HP.

Parathyroid hormone (PTH) is a key regulator of calcium, phosphorus, and bone metabolism. It enhances bone resorption to release calcium into the bloodstream and increases renal tubular calcium reabsorption while promoting the formation of active vitamin D3, thereby indirectly boosting intestinal calcium absorption to elevate serum calcium levels. In HP, insufficient PTH leads to hypocalcemia. Calcium ions play a critical role in myocardial electrophysiology, participating in the plateau phase (Phase 2) of the cardiac action potential. Hypocalcemia prolongs this phase, manifesting on the ECG as QT interval prolongation. It also increases myocardial excitability and automaticity, predisposing patients to various arrhythmias (1). Studies by Hartmann et al. indicate that common cardiovascular manifestations of HP include congestive heart failure and QT interval prolongation (2, 3). In this case, the predominant ECG findings were deep T-wave inversions and QT prolongation. These characteristic changes can closely resemble the ST-T alterations and QT prolongation seen in acute coronary syndrome (ACS) due to myocardial ischemia, particularly when accompanied by angina-like symptoms, which can easily mislead clinicians into diagnosing ACS.

Misdiagnosis often stems from limited clinical reasoning and insufficient awareness of the cardiovascular manifestations secondary to HP. ACS is a common critical condition in cardiology, with chest pain and ECG changes being its hallmark features, prompting clinicians to prioritize this diagnosis. In contrast, the cardiac electrophysiological alterations induced by HP are relatively subtle, and many clinicians lack familiarity with its ECG characteristics and clinical presentations, leading to failure in promptly ordering relevant tests such as PTH, calcium, and phosphorus levels, thereby overlooking the underlying disorder. This case highlights the complex interplay between endocrine disturbances and cardiovascular disease, offering crucial warning and insights for clinical practice.

In this patient, emergency coronary angiography revealed no severe coronary stenosis; however, the possibility of coronary vasospasm should not be overlooked in the differential diagnosis. Coronary artery spasm involves transient, intense contraction of coronary smooth muscle and can occur in arteries with or without atherosclerotic plaques. Its characteristic ECG manifestation is transient ST-segment elevation. In rare cases, atypical presentations such as transient ST-segment depression or T-wave inversion may occur, with rapid ECG normalization upon spasm resolution. This patient's ECG primarily showed QTc prolongation and T-wave inversion without significant ST-segment changes, and it gradually normalized with calcium supplementation, which does not align with the transient ECG pattern typical of coronary vasospasm.

Arterial blood gas analysis revealed: pH 7.5, PaCO_2_ 31 mmHg, PaO_2_ 138 mmHg, and oxygen saturation 99%, indicating acute respiratory alkalosis. When blood pH rises, the binding of calcium ions to albumin increases, leading to decreased ionized calcium levels. This can result in symptoms of increased neuromuscular excitability such as limb numbness, perioral tingling, and muscle twitching, which are clinically easily overlooked manifestations of hypocalcemia. Acute respiratory alkalosis can be alleviated by correcting ventilation and does not require calcium supplementation. In this case, it is undeniable that acute respiratory alkalosis exacerbated the pre-existing hypocalcemic state.

Prompt recognition and correction of misdiagnosis are crucial for improving patient outcomes. In this case, timely ordering of PTH and electrolyte tests allowed for a definitive diagnosis. Adjusting the calcium supplementation regimen and adding oral vitamin D led to rapid symptom relief. Concurrently, ECG abnormalities gradually resolved as serum calcium levels normalized. International studies indicate that cardiac structural and functional impairments due to HP can be reversible with calcium replacement therapy (4–7). However, some case reports have documented no significant recovery of ventricular dilation and systolic function despite calcium supplementation (8, 9).

This case highlights that for patients presenting with chest pain accompanied by dynamic electrocardiographic (ECG) changes—such as T-wave abnormalities and QT-interval prolongation—in conjunction with hypocalcemia, clinicians should maintain a high index of suspicion for endocrine and metabolic disorders after excluding common cardiovascular etiologies. To mitigate the risk of missed or erroneous diagnoses, it is essential for clinicians to broaden their diagnostic approach and promptly obtain relevant laboratory tests, including parathyroid hormone (PTH), calcium, and phosphorus levels, in such cases.

In this case, the precise etiology of the patient's hypoparathyroidism remains undetermined. Thyroid and parathyroid ultrasound revealed a C-TIRADS-4A nodule (approximately 0.85 × 0.77 cm) in the left thyroid lobe. During subsequent follow-up, the patient was referred to a higher-level hospital, where fine-needle biopsy of the thyroid nodule indicated a follicular neoplasm (Bethesda category IV). Surgical excision was recommended for definitive histopathological confirmation; however, the patient declined thyroid surgery. Given the small size of the thyroid follicular lesion and the absence of histopathological evidence supporting compression, invasion, or infiltration of the parathyroid glands by thyroid neoplasia, hypoparathyroidism could not be attributed to the thyroid pathology. The two conditions were considered to coexist incidentally, without a clearly established causal relationship. The patient had no prior history of neck surgery or radiation/chemotherapy. Although the patient has a longstanding history of liver cirrhosis, current liver function tests were normal, and there were no signs of hepatic decompensation. Serum magnesium levels were within normal range, thereby excluding hypomagnesemia as a contributing factor. With regard to other potential etiologies—including autoimmune, genetic, or infiltrative disorders—relevant laboratory tests and investigations were not completed during hospitalization. Consequently, the definitive underlying cause could not be identified.

The patient's subjective experience in this case fully reflects the dual disease burden of HP: impairment from physical symptoms and psycho-emotional distress. Recurrent intermittent respiratory muscle and laryngeal spasms induced intense chest tightness, shortness of breath, and throat constriction, leading to psychological anxiety and disease-related fear. Therefore, clinical management should not solely focus on correcting calcium and phosphorus metabolic parameters but also prioritize the patient's subjective symptoms, psychological state, and long-term quality of life.

In summary, HP-induced angina-like symptoms and ECG evolution are relatively uncommon in clinical practice, but their potential harm should not be underestimated. Through the analysis of this case, we aim to raise clinical awareness of such disorders, enhance differential diagnostic capabilities, prevent misdiagnosis and inappropriate treatment, and thereby provide patients with more accurate and effective healthcare services. Simultaneously, greater attention should be paid to patients’ subjective symptoms, psychological well-being, and long-term quality of life, bolstering health education and psychological intervention, and ensuring regular follow-up to improve long-term patient adherence.

## Data Availability

The original contributions presented in the study are included in the article/Supplementary Material, further inquiries can be directed to the corresponding author.
